# Research on the effectiveness and strategies of new media in promoting voluntary blood donation from a public health perspective in the post-pandemic era

**DOI:** 10.3389/fpubh.2024.1436909

**Published:** 2024-10-14

**Authors:** Jieqiu Weng, Yongzhu Xu, Chengbing Xie, Yunbo Tian, Fang Wang, Ying Cheng

**Affiliations:** Chongqing Blood Center, Chongqing, China

**Keywords:** public health, post-pandemic era, new media, voluntary blood donation, effectiveness, strategy

## Abstract

**Objective:**

This study explores the effectiveness of new media in enhancing public enthusiasm for voluntary blood donation in the post-pandemic era and proposes effective publicity strategies to promote the sustainability and healthy development of blood donation activities.

**Methods:**

A questionnaire survey was widely used to collect public opinions and attitudes toward voluntary blood donation. The sample data underwent rigorous reliability and validity analysis to ensure authenticity and reliability. Statistical methods such as correlation analysis and regression analysis were employed to deeply investigate the underlying relationships between factors like new media publicity, emotional value, social recognition, convenience, information reliability, and willingness to donate blood voluntarily. Based on these analyses, a research model was constructed, and relevant hypotheses were verified through empirical methods.

**Results:**

The study found that new media publicity may be associated with increasing the willingness of the public to voluntarily donate blood. Survey participants indicated that they were more likely to consider donating blood after exposure to new media publicity. Among these factors, the emotional value and content conveyed by the publicity appeared to be particularly important. Additionally, our research revealed that social recognition, the convenience of blood donation, and the reliability of promotional information seemed to have a direct impact on the public’s willingness to donate blood. At the same time, these factors may indirectly promote actual blood donation behavior by enhancing the public’s emotional resonance and acceptance of blood donation.

**Conclusion:**

This study suggests that new media may play multiple positive roles in promoting voluntary blood donation. Based on these findings, we propose a series of strategic recommendations, including further optimizing publicity content, striving to enhance the public’s emotional resonance, improving the reliability of promotional information, and enhancing service convenience. These suggestions aim to potentially raise public awareness and willingness to participate in voluntary blood donation, providing a scientific basis and strong support for the promotion of voluntary blood donation in the post-epidemic era.

## 1 Introduction

The ongoing global public health crisis has presented unprecedented challenges and transformations for voluntary blood donation in the post-pandemic era. As a crucial component of the public health system, voluntary blood donation has been confronted with multiple difficulties during the pandemic, including constrained blood inventory and limited service capacity. The decline in public willingness to participate and diminished safety perception have further exacerbated this issue (1–4). In this context, the effective restoration and enhancement of participation in voluntary blood donation have emerged as critical public health concerns. In this study, new media specifically refers to digital media platforms based on Internet technology, such as social media, online videos, and blogs, which are characterized by fast transmission speed, wide coverage, and strong interactivity. In the post-pandemic era, new media has demonstrated unique advantages in promoting voluntary blood donation. Domestic and international studies have revealed significant impacts of new media on voluntary blood donation. These studies indicate that new media has substantially increased public awareness and participation in voluntary blood donation. Additionally, it has enhanced public trust and recognition of this charitable activity. This enhancement has been achieved through innovative approaches, such as live streaming of blood donation processes and sharing authentic donation narratives (5–7). Research conducted by Padilla-Garrido et al. (8) highlighted the significant impact of innovative and engaging new media promotional content on young people’s willingness to donate blood. The study emphasized that emotional resonance is pivotal in inspiring young people’s enthusiasm for blood donation, with emotional value exerting a significant positive effect on donation intention (8). In other words, emotionally rich and innovative promotional content can substantially enhance young people’s enthusiasm for blood donation. The effective utilization of new media has directly increased blood donation participation rates. In this study, the number of blood donors significantly increased through new media promotion, directly reflecting the notable effect of new media in enhancing blood donation willingness. However, issues such as ensuring information accuracy and protecting donor privacy have also emerged, requiring heightened attention. While new media brings new hope for voluntary blood donation promotion, it also presents a series of challenges. To better leverage this tool, future research should more deeply explore the specific applications and potential problems of new media in voluntary blood donation, thereby providing solid scientific support for the sustainable and healthy development of this charitable cause.

## 2 Materials and methods

### 2.1 Research objects

To ensure the breadth, representativeness, and diversity of the sample, this study adopted stratified random sampling techniques. Survey subjects were randomly selected from various locations in the main urban area of Chongqing, including streets, schools, and communities, to cover people of different ages, genders, education levels, occupational backgrounds, and economic statuses. A total of 2,096 survey questionnaires were distributed, and 2,000 valid questionnaires were recovered, with a questionnaire efficiency rate of 95.42%. This sampling strategy ensured the diversity and representativeness of the sample in terms of demographic characteristics.

#### 2.1.1 Sampling method and sample selection

Stratified random sampling, the key technique adopted in this study, allows us to ensure randomness while fully considering sample diversity. Stratified random sampling involves dividing the population into several strata or subgroups based on one or more characteristics and then randomly selecting samples from each stratum. This method effectively improves sample representativeness and reduces sampling error. First, we determine the stratification criteria. In this study, we stratify based on key characteristics such as age, gender, education level, and occupational background of the respondents. For age, according to the “Blood Donation Law of the People’s Republic of China,” the age range for voluntary blood donors is 18–55 years old. Therefore, we further subdivide this range into four subgroups: 18–25, 26–35, 36–45, and 46–55 years old. For gender, we have two subgroups: male and female. Regarding education level, we categorize respondents based on their highest educational attainment into five subgroups: primary school or below, junior high school, high school/vocational school, college/university, and master’s degree or above. For occupational background, we classify respondents into multiple subgroups such as students, teachers, healthcare workers, civil servants, corporate employees, freelancers, and others, based on their occupation type. We determine the sample size for each stratum based on its proportion in the overall population. For instance, if a particular occupation category comprises a larger proportion of the population, we allocate a correspondingly larger sample size. Within each stratum, we employ random sampling to independently select samples. This approach ensures both randomness in sample selection and adequate representation of each significant subgroup in the sample, thereby enhancing sample representativeness. We pay particular attention to allocating sample sizes reasonably among different strata to reflect their actual proportions in the overall population, which further contributes to the accuracy and reliability of our study findings.

### 2.2 Survey methods and questionnaire content

#### 2.2.1 Survey methods

This survey adopted a questionnaire survey method. Before the survey, we conducted a series of promotional and educational activities to raise public awareness of the importance of blood donation. These activities included collaborating with the media, community outreach, campus events, and online promotion to educate the public about blood donation through various channels, emphasizing its social value and personal health benefits. These activities significantly increased public awareness and interest in blood donation, laying a solid foundation for the subsequent questionnaire survey and actual blood donation activities. The survey was conducted using paper questionnaires. To incentivize respondents to complete the survey, we provided small gifts as a token of appreciation upon completion. Our survey participants were primarily from the 18 to 55 age group to ensure data validity and relevance. We designed a multi-dimensional questionnaire to gather detailed information about willingness to donate blood without compensation. During the survey process, we strictly adhered to ethical principles, ensuring that all participants’ privacy was fully protected. The questionnaires were filled out anonymously, and all collected data was used solely for the purposes of this study. No personal information was disclosed to third parties without the consent of the survey participants.

#### 2.2.2 Questionnaire content

The questionnaire content was divided into two parts: basic information and a scoring scale. The basic information part included gender, age, education level, monthly income (monthly consumption expenditure for students), occupation, and marital status, which were used to comprehensively understand the background information of the respondents. The detailed recording of these demographic characteristics helped analyze the differences in views and attitudes toward voluntary blood donation among different groups. At the same time, the questionnaire also asked whether the respondents had participated in voluntary blood donation and the new media promotion methods they were exposed to, to assess the coverage of promotion channels. Scoring scale: This part used the Likert scale to score different dimensions (such as social recognition, convenience, information reliability, emotional value, publicity content, and willingness to donate blood voluntarily). Through the above questionnaire design, we could comprehensively and in-depth understand the impact of new media publicity on promoting voluntary blood donation, thereby providing a reference for formulating more effective publicity strategies. By establishing a model to find out the promotion strategies of new media for blood donation publicity and actually applying relevant methods for a 6-month experiment, after the end of the experiment, a second survey was conducted on the previously surveyed population through telephone interviews to observe whether new media publicity had a promoting effect on the willingness to donate blood.

#### 2.2.3 Questionnaire design

##### 2.2.3.1 Design logic and theoretical support

The questionnaire design follows the principles of scientific rigor and comprehensiveness, aiming to assess the impact of new media on the willingness to donate blood voluntarily through a multi-dimensional indicator system. The design logic is primarily based on theories such as the theory of planned behavior, social identity theory, information credibility theory, and emotional marketing theory in consumer behavior (9–24). Specifically:

- Social Recognition: Based on social identity theory, four items (A1–A4) were designed to understand the public’s recognition of the social value of voluntary blood donation.
- Convenience: Referring to the concept of convenience in marketing, three items (B1–B3) were designed to examine the impact of the accessibility of blood donation information and the simplicity of the participation process on the public’s willingness to donate blood.
- Information Reliability: Based on information credibility theory, three items (C1–C3) were designed to assess the public’s trust in the content of voluntary blood donation promoted by new media.
- Emotional Value: Drawing on emotional marketing and emotional branding theories, four items (D1–D4) were designed to explore the role of individual emotional experiences in voluntary blood donation behavior.
- Promotional Content: Related to content strategies in advertising and marketing, four items (E1–E4) were designed to evaluate the attractiveness, novelty, and effectiveness of promotional content in enhancing the willingness to donate blood.
- Willingness to Donate Blood Voluntarily: Directly related to the theory of planned behavior, four items (F1–F4) were designed to measure the public’s willingness to donate blood and their willingness to recommend it.

##### 2.2.3.2 Specific questions and reasons for selection

A1: “I feel that participating in voluntary blood donation can contribute to society”: This item aims to measure the public’s recognition of the social value of voluntary blood donation, considering that being able to contribute to society is one of the important motivations for participating in voluntary blood donation.

B2: “I do not need to spend too much time searching for voluntary blood donation information”: This item reflects the impact of the accessibility of blood donation information on the public’s enthusiasm for participation. Convenient information acquisition channels help increase blood donation participation.

C3: “I trust the content promoted by new media about voluntary blood donation”: Trust is key to information acceptance and behavior conversion. This item assesses the public’s trust in the content promoted by new media, which directly affects their willingness to donate blood.

D3: “When I see new media promoting voluntary blood donation, I feel happy about it”: Emotional resonance is an important factor driving behavior. By measuring the public’s positive emotional response to promotional content, their blood donation behavior can be indirectly predicted.

E4: “The content of the new media’s promotion of voluntary blood donation has increased my enthusiasm for blood donation”: This item directly evaluates the promoting effect of promotional content on the willingness to donate blood, which is one of the key indicators for verifying the effectiveness of new media promotion.

### 2.3 Design and implementation of new media strategies

To effectively raise public awareness and participation in voluntary blood donation, this study carefully designed and implemented a series of targeted and highly effective new media strategies. These strategies include specific measures such as optimizing promotional content, sharing real stories, improving information reliability, enhancing service convenience, fully utilizing new media channels, and strengthening social recognition. Optimizing promotional content involves using creative visual designs, information integration and simplification, and emotional resonance to vividly showcase the process and social value of blood donation, thereby enhancing the attractiveness and appeal of the content. Sharing real stories aims to inspire the public’s enthusiasm for blood donation and foster a positive blood donation culture through story collection and display, celebrity endorsements, and interactive feedback. To improve information reliability, we have established close partnerships with authoritative institutions such as health departments and the Red Cross to ensure the authority and accuracy of promotional information. Additionally, we transparently disclose relevant data from blood donation activities to enhance public trust. Enhancing service convenience involves measures such as online booking services, map navigation services, and one-stop services to improve donor satisfaction and loyalty. To fully utilize new media channels, we have opened official accounts on mainstream new media platforms such as Weibo, WeChat, and short video platforms for precise push notifications and interactive marketing, attracting public attention and engaging them in discussions about blood donation. Strengthening social recognition includes establishing a blood donation recognition system, collaborating with mainstream media for widespread reporting and promotion, and encouraging organizations such as businesses, schools, and communities to organize blood donation activities, thereby enhancing the social honor of blood donors. The detailed steps include strategy development, content creation and review, platform publishing and interaction, and data analysis and optimization. We have established a dedicated team responsible for implementing the overall strategy and regularly collecting and analyzing data to optimize our strategies.

### 2.4 Quality control

To ensure data quality, this study adopted multiple quality control measures.

Cronbach’s alpha reliability analysis was used to assess the reliability of the questionnaire data. The reliability coefficients of each dimension were all higher than 0.7, indicating that the data has high reliability.

The KMO and Bartlett’s test of sphericity were used to confirm that the data was suitable for factor analysis. The factor analysis results verified the structural validity of the questionnaire.

### 2.5 Statistical analysis

SPSS 26.0 statistical software was used for data analysis. The analysis steps included descriptive statistics, reliability analysis, validity analysis (including KMO and Bartlett’s test, factor analysis, AVE and CR indicators, and discriminant validity analysis), and model fit verification (through indicators such as χ^2^/df, GFI, RMSEA, RMR, CFI, NFI, and NNFI). The results showed that the model fit was good, ensuring the accuracy and credibility of the analysis results.

## 3 Results

This survey involved a total of 2,000 subjects, with slightly more males than females. The age was mainly distributed between 18 and 35 years old. The education level was mainly college and undergraduate. The monthly income was mainly concentrated in two intervals: 3,000 yuan and below, and 5,001–8,000 yuan. The main occupational groups were students, medical workers, civil servants, and teachers. The unmarried status was slightly higher than the married status. In terms of voluntary blood donation, nearly half of the respondents indicated that they had participated in voluntary blood donation before. See Table 1.

**Table 1:** Basic information of survey respondents (*n* = 2,000).

Category	Option	Frequency	Percentage (%)	Cumulative percentage (%)
Gender	Male	1,073	53.65	53.65
	Female	927	46.35	100.00
Age	18–25 years old	759	37.95	37.95
	26–35 years old	491	24.55	62.50
	36–45 years old	381	19.05	81.55
	Over 45 years old	369	18.45	100.00
Education level	Primary school and below	293	14.65	14.65
	Junior high school	507	25.35	40.00
	High school	171	8.55	48.55
	College and undergraduate	961	48.05	96.60
	Master’s degree and above	68	3.40	100.00
Monthly income	3,000 yuan and below	859	42.95	42.95
	3,001–5,000 yuan	201	10.05	53.00
	5,001–8,000 yuan	674	33.70	86.70
	Above 8,000 yuan	266	13.30	100.00
Occupation	Student	626	31.30	31.30
	Teacher	182	9.10	40.40
	Medical staff	271	13.55	53.95
	Civil servant	238	11.90	65.85
	Military personnel	169	8.45	74.30
	Worker	113	5.65	79.95
	Farmer	171	8.55	88.50
	Enterprise/company employee	108	5.40	93.90
	Self-employed	92	4.60	98.50
	Freelancer	30	1.50	100.00
Marital status	Unmarried	1,013	50.65	50.65
	Married	987	49.35	100.00
Participated in voluntary blood donation	Yes	942	47.10	47.10
	No	1,058	52.90	100.00
Total		2,000	100.0	100.0

This table evaluates the impact of new media on voluntary blood donation through multiple dimensions (social recognition, convenience, information reliability, emotional value, publicity content, and willingness to donate blood voluntarily). Under each dimension, there are a series of specific evaluation indicators. These indicators collectively constitute a comprehensive evaluation of the effectiveness of new media in promoting voluntary blood donation. See Table 2.

**Table 2:** Construction of the indicator system.

Dimension	Code	Evaluation indicator
Social recognition	A1	I feel that participating in voluntary blood donation can contribute to society.
	A2	I feel that participating in voluntary blood donation can achieve a higher level of value.
	A3	I feel that participating in voluntary blood donation can help others.
	A4	I feel a sense of mission when participating in voluntary blood donation.
Convenience	B1	I can see voluntary blood donation promotion content through various channels.
	B2	I do not need to spend too much time searching for voluntary blood donation information.
	B3	To a certain extent, it has increased my enthusiasm for voluntary blood donation.
Information reliability	C1	I believe that the content promoted by new media about voluntary blood donation is accurate.
	C2	I believe that the content promoted by new media about voluntary blood donation is true.
	C3	I trust the content promoted by new media about voluntary blood donation.
Emotional value	D1	I will feel proud of myself for participating in a voluntary blood donation.
	D2	Voluntary blood donation is a noble mission, and I will feel happy.
	D3	When I see new media promoting voluntary blood donation, I feel happy about it.
	D4	When I see negative news related to voluntary blood donation, I feel very angry.
Publicity content	E1	The content of the new media’s promotion of voluntary blood donation is very novel and in line with the public.
	E2	The content of the new media’s promotion of voluntary blood donation is very attractive.
	E3	I really like the innovation in the way new media promotes voluntary blood donation.
	E4	The content of the new media’s promotion of voluntary blood donation has increased my enthusiasm for blood donation.
Willingness to donate blood voluntarily	F1	I am willing to actively participate in voluntary blood donation work.
	F2	New media’s promotion of voluntary blood donation has made me very interested in voluntary blood donation.
	F3	I will recommend relatives and friends watch the new media’s promotion of voluntary blood donation.
	F4	I will recommend that relatives and friends participate in the cause of voluntary blood donation.

The Cronbach’s *α* coefficients for each dimension were at high levels, indicating excellent internal consistency and reliability of the scale, as shown in Table 3.

**Table 3:** Cronbach’s reliability analysis.

Dimension	Items	Cronbach’s *α* coefficient
Social recognition reliability	4	0.827
Convenience reliability	3	0.787
Information reliability	3	0.743
Emotional value reliability	4	0.816
Publicity content reliability	4	0.826
Voluntary blood donation intention reliability	4	0.773

The KMO and Bartlett’s test results (KMO value of 0.942, Bartlett’s test of sphericity approximate chi-square value of 17,182.438, degrees of freedom of 231, and *p*-value of 0.000) indicated that the dataset was highly suitable for factor analysis, as shown in Table 4.

**Table 4:** Validity analysis.

KMO and Bartlett’s test		
KMO value		0.942
Bartlett’s test of sphericity approx.	Chi-Square	17,182.438
	*df*	231
	*p-*value	0.000

The rotated factor loading coefficient table revealed multiple dimensions related to voluntary blood donation, including personal motivations, information dissemination, trust in new media publicity, positive attitudes and recommendation intentions, and the attractiveness of new media publicity, as shown in Table 5.

**Table 5:** Rotated factor loading coefficients.

Name	Factor loading coefficients						Communality (common factor variance)
	Factor 1	Factor 2	Factor 3	Factor 4	Factor 5	Factor 6	
A1		0.757					0.688
A2		0.702					0.606
A3		0.780					0.707
A4		0.764					0.645
B1			0.661				0.659
B2			0.662				0.624
B3			0.695				0.687
C1					0.552		0.679
C2					0.631		0.654
C3					0.714		0.661
D1	0.765						0.716
D2	0.616						0.546
D3	0.660						0.540
D4	0.587						0.561
E1						0.675	0.547
E2						0.791	0.759
E3						0.586	0.518
E4						0.690	0.605
F1				0.637			0.591
F2				0.496			0.540
F3				0.804			0.714
F4				0.785			0.714

Rotation method: Varimax with Kaiser normalization.

The analysis of Pearson correlation coefficients showed that social recognition, convenience, information reliability, emotional value, and publicity content had significant positive correlations with voluntary blood donation intention. Among them, emotional value and publicity content had the strongest correlation. This suggests that improving these factors may help enhance the public’s willingness to donate blood voluntarily, as shown in Table 6.

**Table 6:** Pearson correlation—standard format.

	Mean	Sd	Social recognition	Convenience	Information reliability	Emotional value	Publicity content	Voluntary blood donation intention
Social recognition	3.242	0.912	1					
Convenience	3.280	0.968	0.632**	1				
Information reliability	3.345	0.996	0.512**	0.627**	1			
Emotional value	3.174	0.819	0.424**	0.505**	0.573**	1		
Publicity content	3.136	0.797	0.400**	0.522**	0.510**	0.722**	1	
Voluntary blood donation intention	3.261	0.899	0.424**	0.520**	0.501**	0.530**	0.519**	1

*p < 0.05; **p < 0.01.

**Figure 1:**
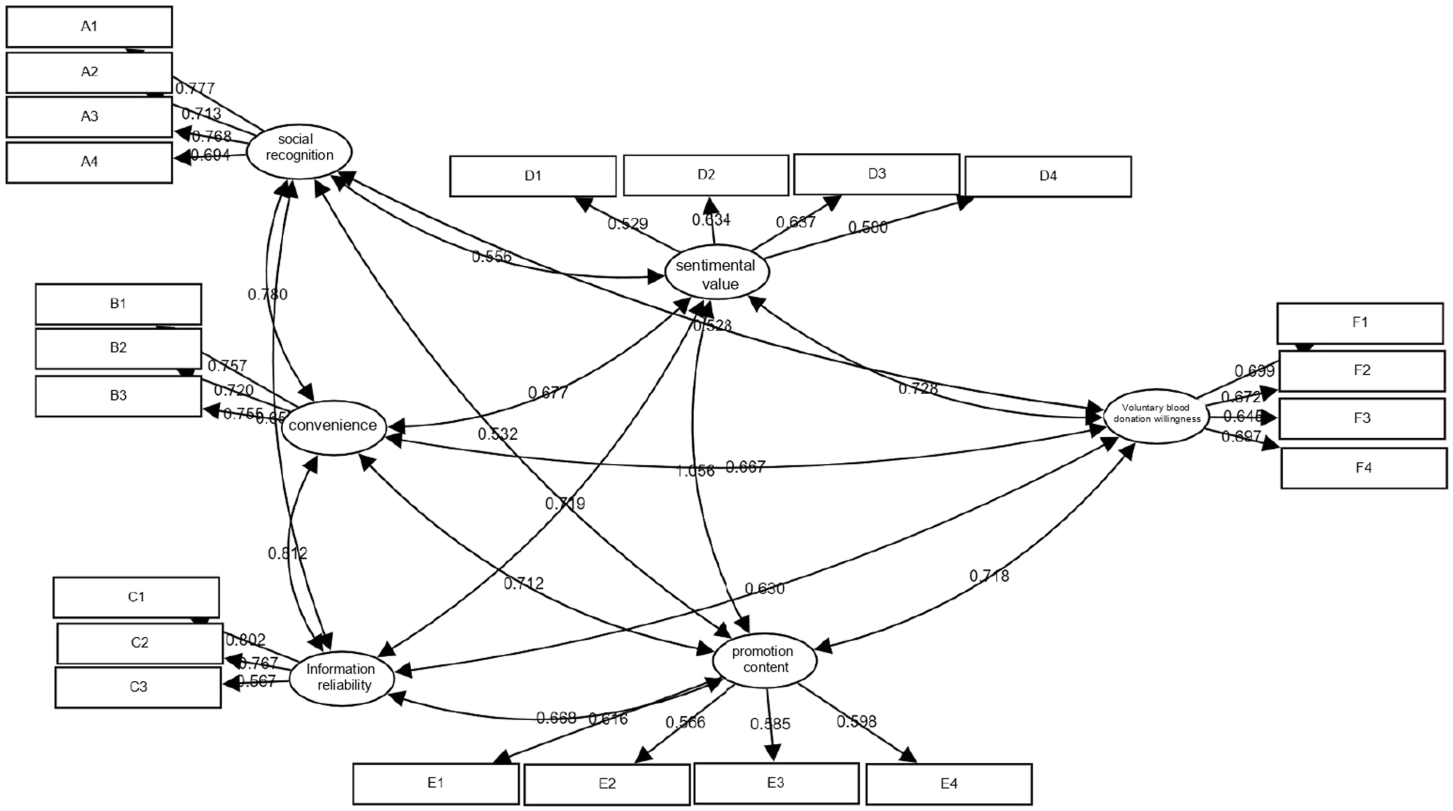
Model diagram.

The results showed that most of the indicators, such as χ^2^/df, GFI, CFI, NFI, NNFI, TLI, AGFI, and IFI, reached or exceeded the judgment criteria, indicating a satisfactory fit between the model and the data. RMSEA and SRMR were also within acceptable ranges, and the 90% confidence interval of RMSEA was stable. Overall, the model fit was good, as shown in Table 7 and Figure 1.

**Table 7:** Model fit indices.

Commonly used indices	χ^2^	*df*	*p*	Chi-square to degrees of freedom ratio (χ^2^/*df*)	GFI	RMSEA	RMR	CFI	NFI	NNFI
Judgment criteria	–	–	>0.05	<3	>0.9	<0.10	<0.05	>0.9	>0.9	>0.9
Value	1,241.716	194	0.000	1.401	0.946	0.052	0.035	0.938	0.928	0.927
Other indices	TLI	AGFI	IFI	PGFI	PNFI	PCFI	SRMR	RMSEA 90% CI		
Judgment criteria	>0.9	>0.9	>0.9	>0.5	>0.5	>0.5	<0.1	–		
Value	0.927	0.929	0.939	0.725	0.779	0.788	0.040	0.049–0.055		

Default Model: χ^2^ (231) = 17,261.553, *p* = 1.000.

Based on the hypothesis test results, new media publicity, social recognition, convenience, information reliability, emotional value, and publicity content significantly influenced the willingness to donate blood voluntarily. The comprehensive optimization of various factors will help improve the public’s enthusiasm for blood donation, as shown in Table 8.

**Table 8:** Hypothesis test results.

Path	*t*	*p*	Hypothesis
New media publicity => Willingness to donate blood voluntarily	30.722	0.000	Supported
Social recognition => Willingness to donate blood voluntarily	4.425	0.000	Supported
Convenience => Willingness to donate blood voluntarily	10.678	0.000	Supported
Information reliability => Willingness to donate blood voluntarily	11.192	0.000	Supported
Emotional value => Willingness to donate blood voluntarily	12.162	0.000	Supported
Publicity content => Willingness to donate blood voluntarily	10.660	0.000	Supported

Social recognition, convenience, and information reliability had indirect and direct effects on the willingness to donate blood voluntarily through emotional value and publicity content, respectively. The total, direct, and indirect effects of all paths were significant, indicating that these paths significantly influenced the willingness to donate blood voluntarily, as shown in Table 9.

**Table 9:** Mediation effect test table.

Item	Significance	Effect value	95% CI		Standard error (SE) value	*z/t* value	*p*-value
			Lower limit	Upper limit			
Social recognition => Emotional value => Willingness for voluntary blood donation Convenience = > Emotional value => Willingness for Voluntary blood donation Information reliability => Emotional value => Willingness for voluntary blood donation	Indirect effect	0.178	0.160	0.203	0.011	16.173	0.000
	Direct effect	0.239	0.200	0.278	0.020	11.993	0.000
	Total effect	0.418	0.379	0.457	0.020	20.916	0.000
Social recognition => Emotional value => Willingness for voluntary blood donation Convenience => Emotional value => Willingness for voluntary blood donation Information reliability => Emotional value => Willingness for voluntary blood donation	Indirect effect	0.168	0.158	0.207	0.012	14.022	0.000
	Direct effect	0.315	0.277	0.352	0.019	16.423	0.000
	Total effect	0.483	0.448	0.518	0.018	27.211	0.000
Social recognition => Emotional value => Willingness for voluntary blood donation	Indirect effect	0.187	0.179	0.235	0.014	13.193	0.000
	Direct effect	0.266	0.227	0.305	0.020	13.264	0.000
	Total effect	0.453	0.418	0.487	0.017	25.903	0.000
Social recognition => Publicity content => Willingness for voluntary blood donation	Indirect effect	0.164	0.145	0.190	0.011	15.171	0.000
	Direct effect	0.254	0.215	0.293	0.020	12.844	0.000
	Total effect	0.418	0.379	0.457	0.020	20.916	0.000
Convenience => Publicity content => Willingness for voluntary blood donation	Indirect effect	0.165	0.153	0.203	0.013	12.824	0.000
	Direct effect	0.318	0.280	0.357	0.020	16.253	0.000
	Total effect	0.483	0.448	0.518	0.018	27.211	0.000
Information reliability => Publicity content => Willingness for voluntary blood donation	Indirect effect	0.164	0.157	0.206	0.012	13.128	0.000
	Direct effect	0.289	0.252	0.326	0.019	15.213	0.000
	Total effect	0.453	0.418	0.487	0.017	25.903	0.000

After the new media publicity, the number of people participating in blood donation increased from 942 in the first questionnaire survey to 1,382 in the second questionnaire survey, while the number of people who had not participated in blood donation correspondingly decreased. This suggests that new media publicity may have contributed to increased blood donation participation.

## 4 Discussion

The survey data presented in the table encompasses populations of diverse genders, ages, education levels, and marital statuses, providing rich and varied data with broad representativeness. Notably, the diversity in monthly income distribution and occupational types offers valuable reference information for an in-depth study of social cognition, attitudes, and behaviors toward voluntary blood donation. Differences in perceptions and participation in voluntary blood donation were observed among groups with varying economic conditions and occupational backgrounds, providing an important basis for developing more precise promotional strategies.

According to the Cronbach’s reliability analysis results in Table 3, each dimension of the scale showed excellent internal consistency. Specifically, the Cronbach’s *α* coefficients for social recognition, convenience, information reliability, emotional value, publicity content, and willingness to donate blood voluntarily were all at high levels, ranging from 0.743 to 0.827. These results indicated that the items used to measure each dimension of the scale had high consistency in assessing the public’s recognition of voluntary blood donation, the convenience of the donation process, the reliability of information, emotional value, the attractiveness of publicity content, and the willingness to donate blood. Although the *α* coefficient for the information reliability dimension was slightly lower, it was still within an acceptable range, ensuring the reliability of the data. Overall, the scale could effectively evaluate the impact of new media on voluntary blood donation activities and the attitudes and willingness of the public, providing a solid data foundation for subsequent statistical analysis.

According to the validity analysis in Table 4, the KMO value was 0.942, close to 1, indicating a strong correlation between variables and high suitability for factor analysis. The Bartlett’s test of sphericity was significant (*p* = 0.000), further confirming the applicability of factor analysis to the dataset. The high KMO value and significant Bartlett’s test indicated that the variables were not independent, and factor analysis could extract meaningful common factors. Factor analysis is a dimensionality reduction technique that can extract a few common factors from multiple variables, and these common factors can explain most of the variation in the original variables. In this study, through factor analysis, we could gain a deeper understanding of the structure behind the data, identify the key factors influencing the willingness and behavior of voluntary blood donation, and provide a scientific basis for the formulation of relevant policies and publicity activities.

Refined Translation:

Factor 1: Intrinsic Motivation and Pride in Voluntary Blood Donation.

This factor primarily encompasses the intrinsic motivations and personal feelings associated with participating in voluntary blood donation. It reflects an individual’s value recognition of voluntary blood donation behavior (A1, A2), the desire to help others (A3), and the resulting sense of mission (A4). Additionally, this factor captures the emotional experiences of individuals after participating in voluntary blood donation, such as feeling proud (D1), feeling happy (D2), and reactions to negative news (D4).

Factor 2: Accessibility of Voluntary Blood Donation Information.

This factor relates to the dissemination and acquisition of voluntary blood donation information. It reflects an individual’s ability to see promotional information about voluntary blood donation through various channels (B1), without spending too much time searching for relevant information (B2). To a certain extent, this information increases an individual’s enthusiasm for voluntary blood donation (B3).

Factor 3: Trust in New Media Publicity Content.

This factor represents an individual’s level of trust in the content promoting voluntary blood donation through new media. It includes the belief that the publicity content in new media is accurate (C1), true (C2), and an individual’s trust in this content (C3).

Factor 4: Positive Attitude and Willingness to Recommend Voluntary Blood Donation.

This factor involves an individual’s positive attitude toward voluntary blood donation and willingness to recommend others to participate. It reflects an individual’s willingness to actively participate in voluntary blood donation work (F1), a strong interest in voluntary blood donation (F2), and the willingness to recommend relatives and friends to watch new media publicity about voluntary blood donation (F3) and participate in the cause of voluntary blood donation (F4).

Factor 5 and Factor 6: Attractiveness of New Media Publicity and Enhancement of Blood Donation Enthusiasm.

These two factors are related to the effectiveness of new media publicity. Factor 5 primarily focuses on the attractiveness (E1, E2) and innovativeness (E3) of new media publicity content, as well as the enhancement of an individual’s blood donation enthusiasm by this content (E4). Factor 6 is associated with trust in new media publicity content and blood donation enthusiasm to some extent.

These dimensions collectively constitute an individual’s comprehensive cognition and emotional attitude toward voluntary blood donation behavior.

Pearson correlation coefficients measure the strength of linear relationships between variables. Firstly, a significant positive correlation exists between social recognition and convenience (correlation coefficient of 0.632**), indicating that as public social recognition of voluntary blood donation increases, people are more likely to perceive blood donation as convenient. This convenience may stem from satisfaction with the setup of blood donation points, the simplicity of the donation process, or recognition of the services and support received after donation. This positive correlation suggests that improving social recognition helps enhance public perception of the convenience of blood donation. Secondly, significant correlations also exist among social recognition, convenience, and information reliability. The correlation coefficient between social recognition and information reliability is 0.512**, while that between convenience and information reliability is 0.627**. This indicates that when the public has higher social recognition of voluntary blood donation, they are more inclined to believe that information about blood donation is reliable. Similarly, when blood donation is perceived as convenient, the reliability of the information is also enhanced. This interconnectedness emphasizes the importance of information transparency and accuracy in improving public attitudes toward blood donation. Emotional value positively correlates with social recognition, convenience, information reliability, and willingness to donate blood voluntarily, with the highest correlation coefficient of 0.722** with promotional content. This suggests that effective promotional content can evoke public emotional resonance, enhance their emotional value identification with blood donation, and thereby increase their willingness to donate blood voluntarily. The enhancement of emotional value may stem from the public’s sense of identification or achievement with blood donation or their recognition of the social value brought by blood donation. Finally, we focus on the correlation between willingness to donate blood voluntarily and other variables. Willingness to donate blood voluntarily has significant positive correlations with social recognition, convenience, information reliability, emotional value, and promotional content. This implies that these factors jointly influence the public’s willingness to donate blood. In particular, promotional content has a correlation coefficient of 0.519** with willingness to donate blood voluntarily, indicating that promotional work plays a crucial role in driving public willingness to donate blood. Through the analysis of the Pearson correlation coefficient table, we can clearly see how factors such as social recognition, convenience, information reliability, emotional value, and promotional content interact and jointly influence the public’s willingness to donate blood voluntarily. The positive correlations between these factors provide valuable insights that, when promoting voluntary blood donation, it is necessary to comprehensively consider these aspects and formulate comprehensive and effective strategies to enhance the public’s willingness to donate blood.

New media promotion appears to have a notable association with enhancing the willingness to donate blood voluntarily (*t* = 30.722, *p* = 0.000). Meanwhile, participants reported that factors such as social recognition, convenience, information reliability, emotional value, and promotional content influenced their willingness to donate blood voluntarily. In particular, social recognition (*t* = 4.425, *p* = 0.000) and convenience (*t* = 10.678, *p* = 0.000) emerged as significant factors that may promote blood donation and enhance the willingness to donate, highlighting the public’s awareness of the social value and convenience associated with blood donation. The importance of information reliability (*t* = 11.192, *p* = 0.000) lies in ensuring the accuracy of blood donation information, which is crucial for increasing the willingness to donate. Emotional value (*t* = 12.162, *p* = 0.000) positively influences blood donation behavior by enhancing the public’s sense of identification and satisfaction. Furthermore, carefully designed promotional content (*t* = 10.660, *p* = 0.000) effectively stimulates the public’s willingness to donate blood. Therefore, to improve the blood donation rate, relevant departments and organizations should comprehensively optimize these aspects, including utilizing new media to expand publicity, enhancing social recognition, providing convenient services, ensuring information accuracy and authority, and focusing on emotional resonance and the attractiveness of promotional content (25–27).

Through a model analysis of blood donation willingness, several key strategies were discovered that can significantly improve the public’s willingness to participate in blood donation. The model analysis showed that an individual’s understanding of the benefits of blood donation is highly correlated with their willingness to donate. Therefore, one strategy is to increase publicity efforts, especially through social media and community activities, to introduce in detail the benefits of blood donation to society and personal health. Relevant departments and organizations should focus on the management and optimization of these aspects. Specifically, new media can be utilized for extensive and effective publicity, enhancing social recognition of voluntary blood donation, providing convenient blood donation services, ensuring the reliability of blood donation information, and strengthening the emotional value of blood donation activities, while carefully designing promotional content to attract and encourage more people to participate in voluntary blood donation. The model also found that to improve the willingness to donate blood, these factors should be comprehensively considered, especially the cultivation of emotional value and the optimization of promotional content (28–30). Furthermore, the model analysis indicated that social recognition, convenience, and information reliability have significant influences on the willingness to donate blood. Based on this, strategies include organizing community blood donation activities, encouraging teams or groups to participate in blood donation together, and utilizing short video challenges, official live broadcasts of the blood donation process, sharing real blood donor stories and experiences, celebrity effects, inviting public figures to participate in blood donation publicity to set an example, and simplifying the blood donation process and improving the blood donation experience, which are also key factors in motivating more people to join the ranks of blood donors.

To verify the corresponding strategies discovered by the model, this study implemented a 6-month new media publicity intervention and conducted a before-and-after comparative survey on the same group of respondents, intuitively demonstrating the significant effectiveness of the publicity intervention. As shown in Table 10, before the implementation of new media publicity, the blood donation participation rate was only 20.35%, while after the intervention, it soared to 59.1%, with an increase of 775 blood donors. Correspondingly, the number of people who did not participate in blood donation decreased from 1,058 to 618, indicating that the campaign effectively stimulated the enthusiasm of potential blood donors. This data strongly supports the study’s conclusion that new media promotion plays a significant role in promoting voluntary blood donation. New media not only raises public awareness and interest in blood donation but also directly translates into increased blood donation behavior, significantly improving participation rates. This data not only strongly demonstrates the positive effect of new media promotion on increasing willingness to donate blood but also fully validates the scientific and practical nature of the research model in this study.

**Table 10:** Survey of blood donation participation after new media publicity.

	Number of blood donors	Number of non-donors	Total
First questionnaire survey	942	1,058	2,000
Second questionnaire survey	1,382	618	2,000

Based on a comprehensive analysis of the results of this study, we further compared them with a broader literature to more comprehensively explore the effectiveness and strategies of new media in promoting voluntary blood donation. This study found that new media publicity can significantly enhance the willingness to donate blood voluntarily, which is consistent with the findings of studies such as Sümnig et al. (31), who pointed out that social media plays a key role in the mobilization and recruitment of blood donors. Through social media platforms, not only can blood donation information be disseminated, but communication among blood donors can also be facilitated, enhancing community cohesion (31). Harrell et al. (32) found through research that social media (such as Facebook, Twitter, etc.) effectively promoted blood donation in countries such as Brazil, India, and the United States. These platforms enhanced the public’s trust and identification with voluntary blood donation through innovative forms such as sharing real blood donation stories and live broadcasting the blood donation process (32).

## 5 Strategies and recommendations

Based on the research findings, this paper proposes a series of strategies to enhance the effectiveness of new media publicity. 1. Optimization of Promotional Content: Design novel, engaging, and visually impactful promotional materials, such as dynamic images and short videos. These materials should detail the important benefits of blood donation to personal health and society, thereby attracting public attention and enhancing recognition of the value of blood donation. 2. Enhancement of Emotional Resonance: Share compelling stories and experiences from actual blood donors. Emphasize the importance of blood donation as a social responsibility and charitable act to evoke public empathy and deepen understanding of the significance of blood donation. 3. Improvement of Information Reliability and Authoritative Sources: Establish close collaborations with health departments, Red Cross societies, and other authoritative institutions to ensure information accuracy and authority. Regular updates of relevant content should be implemented to maintain information timeliness and practicality. 4. Enhancement of Service Convenience and Multi-Channel Promotion: Utilize multiple channels for promotion to facilitate public access to blood donation information. Develop convenient services such as blood donation appointments and navigation. The donation process should be simplified, and a comfortable environment with thoughtful service should be provided to improve the donation experience. 5. Utilization of New Media Platforms: Fully leverage popular new media channels such as Weibo, WeChat, and short video platforms. Employ big data analysis technology for precise information dissemination to expand the coverage and influence of voluntary blood donation promotion (33–35). 6. Strengthening of Social Recognition: Encourage collective organizations such as enterprises, institutions, schools, and communities to conduct blood donation activities. Establish recognition mechanisms to reward units and individuals actively participating in blood donation, fostering a positive donation atmosphere. 7. Innovation in Promotional Methods: Explore the use of virtual reality technology to simulate the blood donation process, allowing the public to experience donation in a safe environment. Interactive experience zones should be set up at donation sites or public places to enhance public willingness to donate through simulated donations and knowledge quizzes. 8. Protection of Donor Privacy: Strictly adhere to laws and regulations in promotional activities. Maintain strict confidentiality of donors’ personal information, and increase public awareness of blood donation privacy protection through targeted promotion.

## 6 Future research directions

Future research should deeply explore new application paths of new media in public health, such as long-term tracking of the sustained impact of new media publicity on willingness to donate blood, comparative effectiveness of cross-cultural communication strategies, and the integration of new technologies (such as VR and AI) with voluntary blood donation publicity. Current research mainly focuses on the effectiveness of new media publicity in the post-epidemic era, with less discussion on the impact during different epidemic stages (such as outbreak and recovery periods). Future research can focus on the changing patterns and characteristics of public willingness to donate blood during different epidemic stages and explore the adaptability and effectiveness of new media publicity in different stages, providing experience and theoretical support for responding to different public health events.

## 7 Conclusion

In summary, the positive role of new media in promoting voluntary blood donation in the post-epidemic era has been widely recognized. By optimizing publicity content, enhancing emotional resonance, improving information reliability, and enhancing service convenience, public awareness and participation in voluntary blood donation can be further improved, providing strong support for the continued promotion of voluntary blood donation and ultimately promoting the healthy development of the public health system.

## Data Availability

The original contributions presented in the study are included in the article/supplementary material, further inquiries can be directed to the corresponding author.
